# Culturally adaptive storytelling intervention versus didactic intervention to improve hypertension control in Vietnam: a cluster-randomized controlled feasibility trial

**DOI:** 10.1186/s40814-017-0136-9

**Published:** 2017-05-01

**Authors:** Hoa L. Nguyen, Jeroan J. Allison, Duc A. Ha, Germán Chiriboga, Ha N. Ly, Hanh T. Tran, Cuong K. Nguyen, Diem M. Dang, Ngoc T. Phan, Nguyen C. Vu, Quang P. Nguyen, Robert J. Goldberg

**Affiliations:** 1 Institute of Population, Health and Development, 18 Alley 132, Hoa Bang Street, Cau giay District, Hanoi, Vietnam; 2 Department of Quantitative Sciences, Baylor Scott &White Health, Dallas, Texas USA; 3 Department of Quantitative Health Sciences, University of Massachusetts Medical School, Worcester, MA USA; 4 Ministry of Health, Hanoi, Vietnam; 5 Department of Pathophysiology–Immunology, Hanoi School of Public Health, Hanoi, Vietnam; 6 Bates College, Lewiston, ME USA

**Keywords:** Hypertension, Blood pressure, Storytelling, Trial, Vietnam

## Abstract

**Background:**

Vietnam is experiencing an epidemiologic transition with an increased prevalence of non-communicable diseases. Novel, large-scale, effective, and sustainable interventions to control hypertension in Vietnam are needed. We report the results of a cluster-randomized feasibility trial at 3 months follow-up conducted in Hung Yen province, Vietnam, designed to evaluate the feasibility and acceptability of two community-based interventions to improve hypertension control: a “storytelling” intervention, “We Talk about Our Hypertension,” and a didactic intervention.

**Methods:**

The storytelling intervention included stories about strategies for coping with hypertension, with patients speaking in their own words, and didactic content about the importance of healthy lifestyle behaviors including salt reduction and exercise. The didactic intervention included only didactic content. The storytelling intervention was delivered by two DVDs at 3-month intervals; the didactic intervention included only one installment. The trial was conducted in four communes, equally randomized to the two interventions.

**Results:**

The mean age of the 160 study patients was 66 years, and 54% were men. Most participants described both interventions as understandable, informative, and motivational. Between baseline and 3 months, mean systolic blood pressure declined by 8.2 mmHg (95% CI 4.1–12.2) in the storytelling group and by 5.5 mmHg (95% CI 1.4–9.5) in the didactic group. The storytelling group also reported a significant increase in hypertension medication adherence.

**Conclusions:**

Both interventions were well accepted in several rural communities and were shown to be potentially effective in lowering blood pressure. A large-scale randomized trial is needed to compare the effectiveness of the two interventions in controlling hypertension.

**Trial registration:**

ClinicalTrials.gov, NCT02483780

## Background

Vietnam is in an epidemiological transition. The overall morbidity and mortality from non-communicable diseases has been rising rapidly over the last two decades in this country and is a major societal problem [1, 2]. The changing profile of chronic disease in Vietnam parallels changes in the socio-demographic characteristics of the population and rapid increases in life expectancy (from 65 years in 1989 to 73 years in 2013) [1–5]. In Vietnam, non-communicable diseases (NCDs) account for a rapidly growing share of all deaths, increasing from 56% in 1990 to 72% in 2010 [6]. Cardiovascular disease is now the leading cause of death in Vietnam, accounting for 30% of all deaths, and hypertension is one of the leading contributors to these death rates [2]. A national survey in eight Vietnamese provinces and cities found that the prevalence of hypertension was 25% in persons 25 years and older, and the frequency of this condition increased with advancing age. The Vietnam National Health Survey estimated that, by 65 years of age, nearly one-half of all Vietnamese men and women will have hypertension [7].

In spite of the economic hardships that exist in Vietnam, inexpensive health care, including generic medications to treat hypertension, are readily available. Our research team has, however, previously reported disconcerting results from a population-based survey of residents of Thai Nguyen province in 2011 in which only one-third of persons diagnosed with hypertension were aware of their condition. Furthermore, of those diagnosed with hypertension, only 43% were on treatment; of those being treated for hypertension, only 39% had achieved appropriate control [8].

Given this background, we developed two different strategies to promote engagement in the care of persons diagnosed with hypertension in rural Vietnam: didactic versus storytelling intervention. In our previous work in the USA, we found storytelling to be a potent intervention technique for improving blood pressure control [9]. According to our conceptual model of storytelling that has been previously published, [10] the power of storytelling follows from being emotionally engaged with a story that resonates with core concepts of culture, including family and daily lived experiences. Across all human cultures, storytelling is a core part of community and family life [11] and, as such, can be used in virtually any setting.

To fill in information gaps that persisted beyond the powerful stories told in the natural voices of our native Vietnamese participants, we added specific didactic content. The second intervention strategy included only this didactic content. In this article, we report 3-month follow-up results about the feasibility, including participant recruitment, retention, and engagement rates and acceptability of these two intervention strategies among middle-aged and older adults with hypertension residing in several rural communities in the Red River Delta region of Vietnam. We also provide data about the acceptability of our study protocols for patient randomization and data collection activities by clinic staff and physicians. We also report potential effects of the interventions on secondary patient-centered outcomes including levels of blood pressure and medication adherence.

## Methods

The Institutional Review Board at the University of Massachusetts Medical School (H00005592) and the Institute of Population, Health and Development in Hanoi, Vietnam (2014/PHAD/UMMS 01-01) approved this study.

The study was conducted by the Institute of Population Health and Development in Hanoi, Vietnam. At the beginning of the study, standard operation procedures were developed and health care workers and study personnel were carefully trained in specific aspects of the study protocol and in carrying out standard operation procedures.

### Study setting

A detailed protocol of this study has been published previously [10]. In brief, the trial was conducted in Hung Yen province in northern Vietnam, which has a population of approximately 1.2 million people, organized into 10 districts and 161 communes. Four communes (Xuan Quan, Viet Hung, Tan Viet, and Bach sam) located in four different districts in Hung Yen province were selected for this feasibility study based on their general representativeness to the rural northern Vietnamese population.

### Development of storytelling intervention

Cultural adaptation is at the very core of our approach to developing storytelling interventions. The development of the study intervention was based on successfully used protocols developed by our team in the USA for story elicitation, review, editing, and packaging [9]; however, all content for the storytelling intervention was based on our local Vietnamese participants speaking naturally about their condition intersected with their personal and cultural beliefs. The storytelling intervention centered on stories about living with hypertension, with patients speaking in their own words. The development of the storytelling intervention began with six story development groups whose purpose was to (1) gather critical data to inform intervention content, (2) identify patients who would serve as our storytelling “stars,” and (3) develop customized interview guides for the subsequent videotaping of each star. Each story development group had between six and eight participants with newly diagnosed or long-standing hypertension. These video stars had positive experiences in controlling their hypertension and were particularly eloquent and persuasive advocates.

“We Talk about Our Hypertension” focused only on the more optimal management of hypertension, through the use of, and increased adherence to, antihypertensive medications as well as personal lifestyle changes, in which patients told personal stories about how they were able to effectively manage their hypertension. Digital video sequences were rated by a team which included several community members, health care staff, and study staff who viewed and rated the DVDs using a simple questionnaire for strength of content and emotional engagement. Then the most highly rated stories were integrated into two interactive DVDs. Each study DVD included five stories in Vietnamese, the national formal language that all people in Hung Yen province speak, and was approximately 50 min in length.

The domains of the DVDs were organized around stories about the health consequences of hypertension as a silent killer, overcoming barriers to hypertension control, and the importance of adherence to prescribed medication, quitting smoking, dietary changes, weight loss, reducing sodium/salt intake, increasing levels of physical activity, and moderate use of alcohol. Patients’ perceptions about the benefits and use of traditional and Western medicine for managing high blood pressure were also discussed. For example, one of the stars shared the following “*I was diagnosed with both hypertension and diabetes. So the doctors prescribed me two types of medications, one for hypertension and the other for diabetes. From then on, I always take my medications correctly, according to what the doctor had prescribed. Aside from western medicine, I also take some traditional medicine made from bitter melon, dried lotus leaves or gripeweeds every day, perhaps even more so than before*”. Another star shared the following “*Besides hypertension*, *I was also diagnosed with hyperlipidemia and fatty liver so the doctor prescribed me other medications to help me control those conditions as well. However, I also take some traditional medicine such as gripeweed and lotus leaves. Using those two as supplements to treat my conditions makes me feel much better. Having multiple conditions such as hypertension, hyperlipidemia and fatty liver disease, I manage my own medication use”.*

The stories were supplemented by didactic “Learn More” content, which was coordinated with specific patient stories to fill in gaps not covered by the storytellers. The Learn More section included such topics as the following, in lay language: What is hypertension? What are the consequences of untreated hypertension? How may hypertension be treated without medications? Why is it important to take your medication even when you are feeling well? And, how should I speak to my doctor about high blood pressure?

### Development of the didactic intervention

All didactic material was presented in the “Learn More” style as described above. The DVD for the didactic intervention included general recommendations for controlling non-communicable diseases including the importance of having a healthy diet, participation in regular physical exercise, quitting smoking tobacco and drinking less or no alcohol, and need for regular examination checkups; many of the promoted healthy lifestyle changes were directly relevant for lowering blood pressure. In addition, the DVD for patients randomly assigned to this group contained information about other non-communicable diseases other than hypertension. We did not include a true control group without any intervention since the didactic material included only general recommendations for non-communicable diseases which is currently advertised in the mass media in Vietnam.

### Translation of survey instruments

When possible, measures for self-reported study variables were taken from well-validated surveys. We have described in detail the translation process for various study instruments in our previous publication [10]. In brief, our translation team consisted of the two Co-Principal Investigators from Vietnam, who are fluent in the language, the Principal Investigators in the USA, who bring important expertise about hypertension and the intervention approach, and an expert in psychometrics. The translation process involved the following iterative steps: prepare, translate, pretest, revise, and document.

### Study sites

Four communes (Xuan Quan, Viet Hung, Tan Viet, and Bach) in four separate districts were selected for this feasibility trial. Each of the selected communes satisfied the following criteria: (1) had a community health center with a medical doctor, (2) were not previously or currently participating in other studies to improve cardiovascular disease risk management, and (3) had a minimum geographic separation of 12 km (7 miles) from all other study communes to minimize possible contamination.

### Participant eligibility

To be enrolled in this feasibility study, consenting adult men and women needed to fulfill each of the following criteria: (1) be a resident of the selected commune, (2) be aged 50 years or older, (3) have a formal medical diagnosis of hypertension using criteria from the 8th Joint National Commission of High Blood Pressure (Joint National Committee), [12] (4) have blood pressure measures consistently elevated at two time points (screening and at 2 weeks after screening), (5) not be cognitively impaired (as assessed by study physicians according to a standard clinical protocol issued by the Vietnam Ministry of Health), (6) not be a “storyteller” who was used to develop the trial intervention, and (7) not be a family member of another participant in the study. We restricted our patient population to persons 50 years and older since their prevalence of hypertension is greatest, they are often interested in storytelling which was emphasized in the story development groups, and they face unique challenges to managing their blood pressure.

### Study recruitment and randomization

Residents with hypertension from the four eligible communes were randomly assigned to either the storytelling or didactic intervention arms. Participant assignment to the respective study arms was based on the randomization status of their respective commune. Sampling frames that comprised all adult community members were obtained from lists maintained by the local government. Based on these comprehensive resident commune lists, adults 50 years and older were invited to our hypertension screening program. At the initial trial visit, blood pressure measurements were taken and researchers explained the study protocol to possible participants and verbal consent was obtained. Persons who screened positive for hypertension and were not willing to participate in the feasibility trial were referred for usual care and their BP readings were shared with their physicians at local clinics. A second screening visit was scheduled 2 weeks later, at which time blood pressure was re-assessed, and written informed consent was obtained.

### Intervention delivery

We chose to deliver the intervention by digital video disc (DVD) based on a pilot data from a previous survey conducted in the same study setting, which explored the willingness of participating in clinical trials examining the impact of health behavior interventions on improving hypertension control, and the appropriate modes of intervention delivery. Based on this survey (*n* = 84), 92% of surveyed participants expressed that they would like to receive health messages via DVDs (unpublished data).

After obtaining informed consent, a trained community health worker introduced and explained the DVD to the patient and provided instruction in its use. Participants initially viewed the first DVD installment at their local health center and then engaged in a post-media interview and problem-solving session with a community health worker. After the clinic viewing, the patient took the DVD home for subsequent review and shared it with family and friends.

At 3 months after trial randomization, a second installment of the DVD was delivered for home viewing for patients randomized to the storytelling arm. After the second viewing, a study visit was scheduled for a “post-media” interview and re-measurement of blood pressure by a trained community health worker. Patients assigned to the didactic intervention arm only received a single DVD during a clinic visit.

### Participant follow-up

To minimize losses to follow-up and missing data, 1 week before the scheduled follow-up visit local staff contacted participants either by phone or through a home visit to remind them about their subsequent clinic visits. For participants who missed the follow-up visit, study staff came to their homes to interview the study subject and measure their blood pressure within 2 weeks of a scheduled follow-up visit. Local study staff called participants every 2 weeks to find out if participants needed any technical support for using the DVD players and encouraged them to view the standardized DVDs more frequently to improve their adherence to the study intervention. Participants received a DVD player at no cost at the beginning of the study and an Omron blood pressure monitor when they finished the study to support their initial and continued participation in the trial.

### Data collection and management

Data were collected for both study arms using a paper data collection form at the study sites and were subsequently entered into a REDCap online-database (https://ncats.nih.gov/expertise/clinical/redcap) at the Institute of Population Health and Development, by trained research staff. Data were collected at the time of baseline trial enrollment and subsequent follow-up visit at the participants’ local community health center at 1 and 3 months after randomization. Study participants’ levels of blood pressure were measured at each study visit according to a standardized protocol developed by the World Health Organization, as follows. Trained community health workers used a calibrated Omron-automated monitor to measure participant’s blood pressure, and the average of the last two of three readings was entered into the database. Height and weight were measured in the absence of shoes and heavy clothing while waist and hip size were measured by placing the tape horizontally around the smallest part of the waist and the widest portion of the hips, respectively. Data on medication adherence were collected using an adapted eight-item scale for Vietnamese patients with hypertension, with greater scores indicating worse adherence [13, 14].

Trained research nurses at each of the local community health centers collected data on study participant’s socio-demographic factors and risk factors for cardiovascular disease including self-reported tobacco use, alcohol consumption, salt intake, and physical activity using the World Health Organization STEPS questionnaires, which have been validated and used in previous studies examining risk factors for chronic disease among rural Vietnamese adults [15–18]. We ascertained self-reported engagement with the DVDs, including total viewing time in minutes, specific segments that were viewed, and whether the DVD was shared with family or friends.

### Study outcomes

The trial’s feasibility outcomes included participant recruitment, retention, and engagement rates. Recruitment rates were calculated from the number of patients approached and reasons for ineligibility and non-participation. Retention rates were calculated from the number of patients successfully followed up by recording the number and reasons for failure to complete the follow-up assessment. Intervention engagement, which was ascertained from the patient survey as described above, included time spent watching the DVD, satisfaction with the viewing experience, and whether the DVDs were attractive, informative, reasonable in length, and effectively encouraged them to change their current lifestyle practices.

Secondary patient-centered outcomes included levels of systolic and diastolic blood pressure and the proportion of participants with controlled blood pressure defined as in Joint National Committee JNC 8 (<150/90 mmHg for those ≥60 years old and <140/90 mmHg for those <60 years old or with diabetes or chronic kidney disease) [18]. Based on the adapted medication adherence scale and because of small sub-sample sizes, adherence was classified into two categories: moderate to high (0–2) or low (3–8).

In addition, the acceptability of study protocols for randomization and data collection by clinic staff and physicians were assessed through interview and group discussions with these individuals.

### Data analysis

Categorical data were presented as frequency distributions, and continuous variables were presented as medians (Inter quartile range-IQR). By design, we lacked adequate power to detect differential changes over time in the secondary patient-centered outcomes between the two study groups; therefore, we provided 95% confidence intervals for change in these outcomes separately for each of the two study groups.

We used generalized linear mixed regression models which included group assignment, time of assessment, and the interaction between group and time to estimate changes over time in the secondary study outcomes, accounting for clustering of patients within commune and repeated within-person measurements. All analyses were performed using STATA 14.0 (StataCorp. TX).

### Sample size

Leon, Davis, and Kraemer state that “power analyses should not be presented in an application for a pilot study that does not propose inferential results” [19]. Instead, we based our sample size on accepted practice for pilot studies, the considerable experience of the research team, and practical considerations [19, 20]. We aimed to recruit 100 patients in this feasibility study.

## Results

### Intervention development

**Fig. 1:**
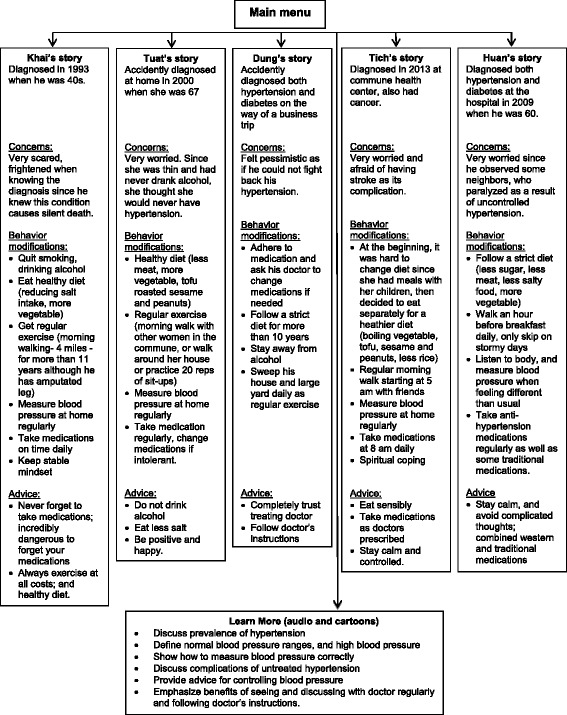
Construct map of the first intervention DVD

A total of six story development groups were conducted. These groups consisted of 36 patients with hypertension who had their blood pressure controlled. The mean age for these story development group participants was 61 years (SD 8.6) and one-half were men. Based on the ensuing group discussion, we selected five individual “stars” who had successfully controlled their blood pressure during the last year and told compelling stories about how they modified their lifestyle practices for better blood pressure control. For those randomly assigned to the storytelling intervention group, two DVDs were produced (Fig. 1). For the group randomized to the didactic intervention arm, a single DVD was produced which included the “Learn More” module about common non-communicable diseases, but did not include stories about hypertension.

### Recruitment, retention rates, and population characteristics

**Fig. 2:**
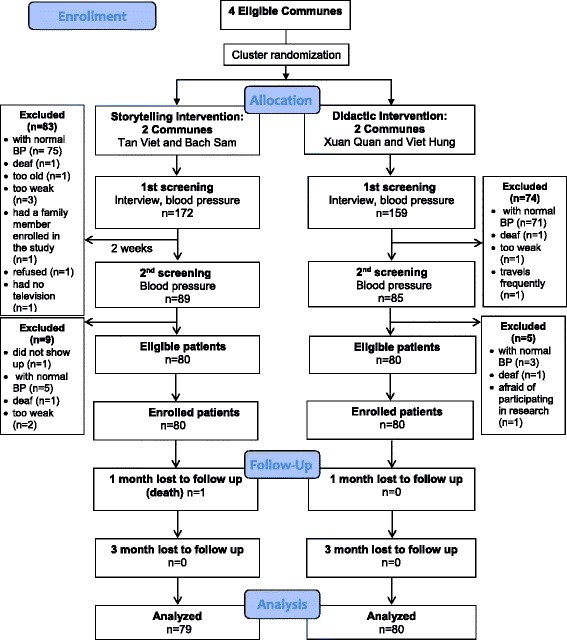
CONSORT flow diagram for the feasibility cluster-randomized controlled trial

Based on the community rosters described previously, 331 patients (172 in the storytelling intervention, 159 in the didactic intervention) who resided in the four selected communes (Xuan Quan, Viet Hung, Tan Viet, and Bach San) were selected for the first screening, which included blood pressure measurement and additional assessment for study eligibility criteria (Fig. 2). After the first screening, 174 patients (89 in storytelling intervention, 85 in the didactic intervention) met our study criteria and were selected for the second screening, which was carried out 2 weeks after the initial screening. After the second screening, 160 eligible patients (80 patients in each group) were identified and invited to participate in the trial. All eligible patients agreed to study participation and provided written informed consent. At 1 month after trial enrollment, all patients were successfully followed up (97% in the clinic and 3% at the patient’s home). At 3 months after enrollment, 99% (159 out of 160) of patients were successfully followed up (94% in the clinic and 6% at the patient’s home). Full details about the screening and follow-up process are provided in Fig. 2.

### Baseline participant characteristics

Full baseline descriptions of study participants are provided in Table 1. The mean age of the study population was 66 years and 54% were men. There were no important differences in study baseline characteristics for those randomized to either the storytelling or didactic intervention arms.

**Table 1:** Patient characteristics at trial enrollment

	Storytelling	Didactic
	Intervention	Intervention
	(*n* = 80)	(*n* = 80)
Age (SD), mean, years	64.6(9.9)	66.9(8.9)
BMI (median-IQR), kg/m^2^	23.1(21.9–24.8)	24.0(21.7–25.5)
Number of adults residing in the household	3(2–4)	3(2–4)
	(*n*,%)	(*n*,%)
Male	39(48.8)	47(58.8)
Ethnicity		
Kinh	80(100)	80(100)
Marital status		
Never married	2(2.5)	0(0)
Currently married	64(80.0)	73(91.2)
Widowed	14(17.5)	7(8.8)
Education levels		
Primary school	21(26.3)	24(30.0)
Secondary school	36(45.0)	38(47.4)
High school	17(21.3)	7(8.8)
College/University	6(7.4)	11(13.8)
Working status		
Employee	3(3.8)	4(5)
Self-employed	12(15)	10(12.5)
Homemaker	11(13.8)	8(10)
Retired	16(20)	26(32.5)
Other	38(47.4)	32(40)
Current smoker	13(16.3)	12(15)
Self-reported CVD comorbidity		
Elevated blood glucose/diabetes	11(18.6)	12(21.8)
Elevated blood cholesterol	34(61.8)	27(52.9)
AMI/angina/stroke	27(33.8)	30(37.5)

*SD* standard deviation, *IQR* interquartile range, *CVD* cardiovascular disease

### Intervention acceptability and engagement

At the time of the 1-month in-person follow-up visit, a survey about the viewing of the first DVD was administered for both study groups. In the storytelling intervention group, the median frequency of viewing the DVD on a weekly basis was two times (IQR 1–4), and the median duration of time spent watching the DVD each time it was reviewed was 38 min (IQR 25–60 min). The majority (79%) of patients in this group reported that they had watched all five “star” stories on the first DVD (Table 2). Each person stated that they understood the words used in the storytelling DVD and that the stars’ stories were culturally acceptable. The majority of patients also reported that the content of the DVD’s was attractive (96%), informative (98%), reasonable in length (86%), and effectively encouraged them to change their lifestyles (99%).

**Table 2:** Post DVD viewing survey by study group

Responses	Storytelling intervention	Didactic intervention
Median (IQR) frequency of viewing DVD per week	2(1–4)	2(1.5–5.5)
Median (IQR) duration of viewing DVD each time (minutes)	38(25–60)	15(7–20)
Viewed all 5 stories (*n*, %)	63(79)	NA
Understandable words (*n*, %)	80(100)	79(98.8)
Culturally acceptable (*n*, %)	80(100)	80(100)
Attractive images (*n*, %)	77(96.3)	67(85.9)
Telling story/health topic attractively (*n*, %)	76(95.0)	67(84.8)
Encouraged to change habits (*n*, %)	78(98.7)	56(70.0)
Length (*n*, %)		
Reasonable	69(86.2)	33(41.2)
Too long	10(12.5)	0(0)
Too short	1(1.3)	47(58.8)
Information (*n*, %)		
Enough	78(97.4)	57(71.3)
Too much	1(1.3)	0(0)
Not enough	1(1.3)	23(28.7)

*NA* not applicable, *IQR* interquartile range

For the didactic intervention group, the median frequency of viewing the DVD on a weekly basis was also two times (IQR 1.5–5.5), and the median duration of viewing the DVD was 15 min (IQR 7–20 min). Most of the patients in this group stated that the didactic instruction DVD, which included only a single “Learn More” module for common non-communicable diseases, was attractive (Table 2). One third of the patients stated that the “Learn more module” did not effectively encourage them to change their lifestyle practices. More than one-half of the subjects in this group reported that the DVD was too short, and nearly one-third stated that there was inadequate information presented about how they might better control their levels of blood pressure.

### Secondary patient-centered outcomes

Our exploratory study outcomes are presented in Tables 3 and 4. Mean systolic and diastolic blood pressure readings improved over time for both study groups (Table 4). However, greater reductions in average blood pressure levels were consistently noted for persons randomly assigned to the storytelling versus the didactic intervention. Of note, this study was not specifically powered to detect statically significant differential changes over time in blood pressure findings for the two study groups.

**Table 3:** Study outcomes for patients assigned to storytelling and didactic interventions

	Storytelling	Didactic
	(*n* = 80)	(*n* = 80)
Systolic blood pressure (mean, SD), mmHg		
Baseline	150.4 (17.5)	144.3 (8.9)
Month 1	139.8 (15.8)	136.6 (20.4)
Month 3	142.2 (17.6)	138.9 (17.5)
Diastolic blood pressure (mean, SD), mmHg		
Baseline	91.3 (9.3)	87.8 (8.1)
Month 1	88.4 (11.0)	85.5 (13.6)
Month 3	85.3 (10.3)	82.7 (13.4)
Controlled blood pressure (%)^a^		
Baseline	16.3	25.0
Month 1	36.3	51.3
Month 3	41.8	48.8
Moderate to high medication adherence (%)^b^		
Baseline	62.3	72.8
Month 3	77.5	57.4

^a^Based on guidelines from the Joint National Commission for the Management of High Blood Pressure in Adults

^b^Based on self-reported items from an adapted medication adherence scale for Vietnamese patients with hypertension

**Table 4:** Improvement over time (95% CI) in study outcomes after exposure to storytelling and didactic interventions

	Storytelling	Didactic
	(*n* = 80)	(*n* = 80)
Systolic blood pressure (mean), mmHg		
Δ Baseline—Month 1	10.6 (6.4–14.8)	7.7 (3.5–11.9)
Δ Baseline—Month 3	8.2 (4.1–12.2)	5.5 (1.4–9.5)
Diastolic Blood Pressure (mean), mmHg		
Δ Baseline—Month 1	3.0 (0.3–5.6)	2.3 (−0.3–4.9)
Δ Baseline—Month 3	6.0 (3.6–8.4)	5.1 (2.7–7.5)
Controlled Blood Pressure (%)^a^		
Δ Month 1—Baseline	19.2 (8.4–30.0)	26.7 (14.9–38.5)
Δ Month 3—Baseline	25.5 (13.0–38.0)	23.4 (10.8–36.6)
Moderate to High Medication Adherence (%)^b^		
Δ Month 3—Baseline	15.8 (0.2–30.0)	−15.4 (−29.1–1.6)

Based on generalized linear models that account for clustering of patients within commune and repeated measurement nested within patients. Small discrepancies from obtaining difference directly from Table 3 are attributable to missing data and numerical estimation algorithms

^a^Based on guidelines from the 8th Joint National Commission for the Management of High Blood Pressure in Adults

^b^Based on self-reported items from an adapted medication adherence scale for Vietnamese patients with hypertension

At 3 months after trial randomization, mean systolic blood pressure had improved by 8.2 mmHg (95% CI 4.1–12.2 mmHg) in the storytelling intervention group and by 5.5 mmHg (95% CI 1.4–9.5 mmHg) in the didactic intervention group; the reduction in the storytelling intervention group was greater compared with that in the didactic intervention (2.7 mmHg; 95%CI −3.0; 8.4 mmHg). Similarly, rates of blood pressure control improved over time for both groups after exposure to the two interventions. The improvement in blood pressure control from pre-intervention to one month follow-up visit was greater for those in the didactic intervention group. At the time of the 3-month follow-up visit, however, the storytelling intervention showed greater overtime improvement in blood pressure control. Self-reported blood pressure medication adherence rates improved for the storytelling intervention group by approximately 15 percentage points, but worsened for the didactic intervention group by an almost similar amount. At 3 months after trial randomization, the improvement gap in medication adherence for the two study groups was 31.2% (95% CI 11.4–15.1), favoring the storytelling intervention group (Table 4).

#### Qualitative findings

During the study follow-up visits, patients shared their thoughts about the value of medication adherence and personal strategies used to achieve adherence. For example, one of the patients in the storytelling intervention group shared the following: “*Prior to study enrollment, I had already exercised regularly and followed a healthy diet. However, I often forgot to take medications on time since I raise a lot of rabbits for living, and have to take care of them. After watching the DVD, I asked my grandson to set up the alarm in my cell phone to remind me to take medications on time even if I am in the middle of doing something*”. Another patient shared the following: “*Thanks to participating in this study, I understand that it is very important to take and adhere to antihypertensive medications. Currently, I take medications every day at 7 am, and never forget*”. In addition, patients also shared their opinions about the impact of the general recommendations on healthy diet, regular physical exercise, and quitting tobacco smoking and alcohol drinking on their health behaviors. For example, one of the patients in the didactic group shared the following comment: “*Previously I drank a lot of alcohol (300-500 ml/day), and did not care about my health. However, since I watched the DVD, I have tried to quit drinking alcohol. Currently, I drink only 1-2 small cups daily (30 ml/cup), and aim to quit completely very soon*”. Another patient shared the following: *“I watched the DVD every day. I also invite my neighbors to come to my house to watch it with me and we try to follow the DVD recommendations”.*

## Discussion

Middle-aged and older adults with hypertension who resided in several rural communities in Vietnam face unique challenges in managing their BP. For example, they are more likely to rely on younger family members to make health decisions, to use alternative approaches to manage their medical conditions, and are less likely to have access to, and use of, technology. We, therefore, developed two literacy sensitive interventions for this population group with one intervention incorporating stories told in the patients’ own voice and the other intervention relying totally on didactic content. Although both interventions showed preliminary evidence of effectiveness in improving hypertension control, rates of improvement in blood pressure were greater for persons who received the storytelling intervention as were improvements in medication adherence.

The results of this feasibility trial also indicated that both the storytelling and didactic interventions were well received among residents of several rural communities in northern Vietnam; although, patient ratings were consistently higher for the storytelling intervention. Most of the patients stated that the storytelling DVDs were understandable, culturally acceptable, informative, attractive, reasonable in length, and effectively encouraged them to change their lifestyle practices. Study protocols for randomization and data collection worked well and were well accepted by patients and clinic staff and physicians.

The storytelling intervention improved medication adherence in the intervention group only. This is likely because many of the stories focused on the value of medication adherence and personal strategies used to achieve adherence. In contrast, the didactic intervention only addressed medication adherence in a generic manner. While medication adherence did not improve in the group randomized to the didactic intervention, reductions in blood pressure were nonetheless observed. One possible explanation is that the general recommendations on healthy diet, regular physical exercise, and quitting tobacco smoking and alcohol drinking may have had a considerable impact on health behavior in this population.

These findings are in agreement with a previous study conducted by our group in Alabama, USA. In this study, we found that exposure to a similar storytelling intervention produced significant reductions in blood pressure for a population of African American patients with hypertension treated at an urban, safety-net setting [9]. To develop the Vietnam storytelling intervention used in the present study, we drew upon the conceptual underpinnings of our previous work [21, 22] and adapted the specific implementation to the new cultural setting. Our conceptual model of storytelling intervention holds that powerful stories told in the patients’ own voice create emotional resonance; through emotional resonance, the viewer is transported into the story and becomes open to change [23, 24]. Although we did not power the study to detect significant changes in blood pressure between the two study groups, it is impressive that the intervention produced statistically significant improvements within each group. Therefore, we believe that the results presented in the paper support the assertion that storytelling is a universally shared and valued human activity that offers many untapped opportunities for effective health intervention.

There are some potential study limitations that must be kept in mind in interpreting our study results. First, this pilot study was intentionally not powered to compare changes over time in the levels of blood pressure between the two study groups because of its design nature. Additionally, due to the small study sample size, we were not able to perform subgroup analyses according to patient’s sex, stage of hypertension, and/or blood pressure status (controlled versus uncontrolled). A full-scale trial with a lager sample size will provide more insights into the efficacy of the intervention in various patient subgroups. Second, there is the potential for contamination despite the distance between adjacent communes being at least 12 km (7 miles). Third, medication adherence was only measured by patient self-report and not by more objective measures, and this paper only included preliminary results up to 3 months of follow-up. Finally, we cannot rule out the fact that some of the improvements in blood pressure and self-reported medication adherence were a result of the collateral benefit from being enrolled and followed up in the context of a clinical trial as opposed to representing a specific intervention effect. Part of the lesser effectiveness of the didactic intervention could have resulted from fewer DVD installments.

## Conclusions

Our preliminary results suggest that both the storytelling and didactic interventions hold potential for improving blood pressure control, but the efficacy of the storytelling intervention was greater. In addition, both interventions were highly rated by our community-based sample of patients. We also documented the feasibility of randomization and data collection efforts in this population. These findings suggest that the implementation of a subsequent large-scale, fully powered, cluster-randomized controlled trial comparing the long-term efficacy of both interventions is an appropriate next step. Results from the full-scale trial will provide health policy makers with practical evidence on how to combat a key risk factor for cardiovascular disease using a feasible, sustainable, and cost-effective intervention that could be used in other developing countries. Although the storytelling intervention holds great promise across a range of cultures, conditions, and economic settings, the present work offers new insights about how this technique may be implemented and developed in low-to-middle income countries.

## Abbreviation

DVD: Digital video disc
