# Erratum to: Culturally adaptive storytelling intervention versus didactic intervention to improve hypertension control in Vietnam: a cluster randomized controlled feasibility trial

**DOI:** 10.1186/s40814-017-0149-4

**Published:** 2017-06-15

**Authors:** Hoa L. Nguyen, Jeroan J. Allison, Duc A. Ha, Germán Chiriboga, Ha N. Ly, Hanh T. Tran, Cuong K. Nguyen, Diem M. Dang, Ngoc T. Phan, Nguyen C. Vu, Quang P. Nguyen, Robert J. Goldberg

**Affiliations:** 1 Institute of Population, Health and Development, 18 Alley 132, Hoa Bang, Street, Cau giay District, Hanoi, Vietnam; 2 Department of Quantitative Sciences, Baylor Scott & White Health, Dallas, Texas USA; 3 Department of Quantitative Health Sciences, University of Massachusetts Medical School, Worcester, MA USA; 4 Ministry of Health, Hanoi, Vietnam; 5 Department of Pathophysiology–Immunology, Hanoi School of Public Health, Hanoi, Vietnam; 6 Bates College, Lewiston, ME USA

## Erratum

Following publication of the original article [1], The author found that there was a minor error in Fig. 2 that needed to be corrected. In the Storytelling intervention group, one patient lost to follow up should be at 3 month, not at 1 month. The corrected Fig. 2 is included below.

**Fig. 2:**
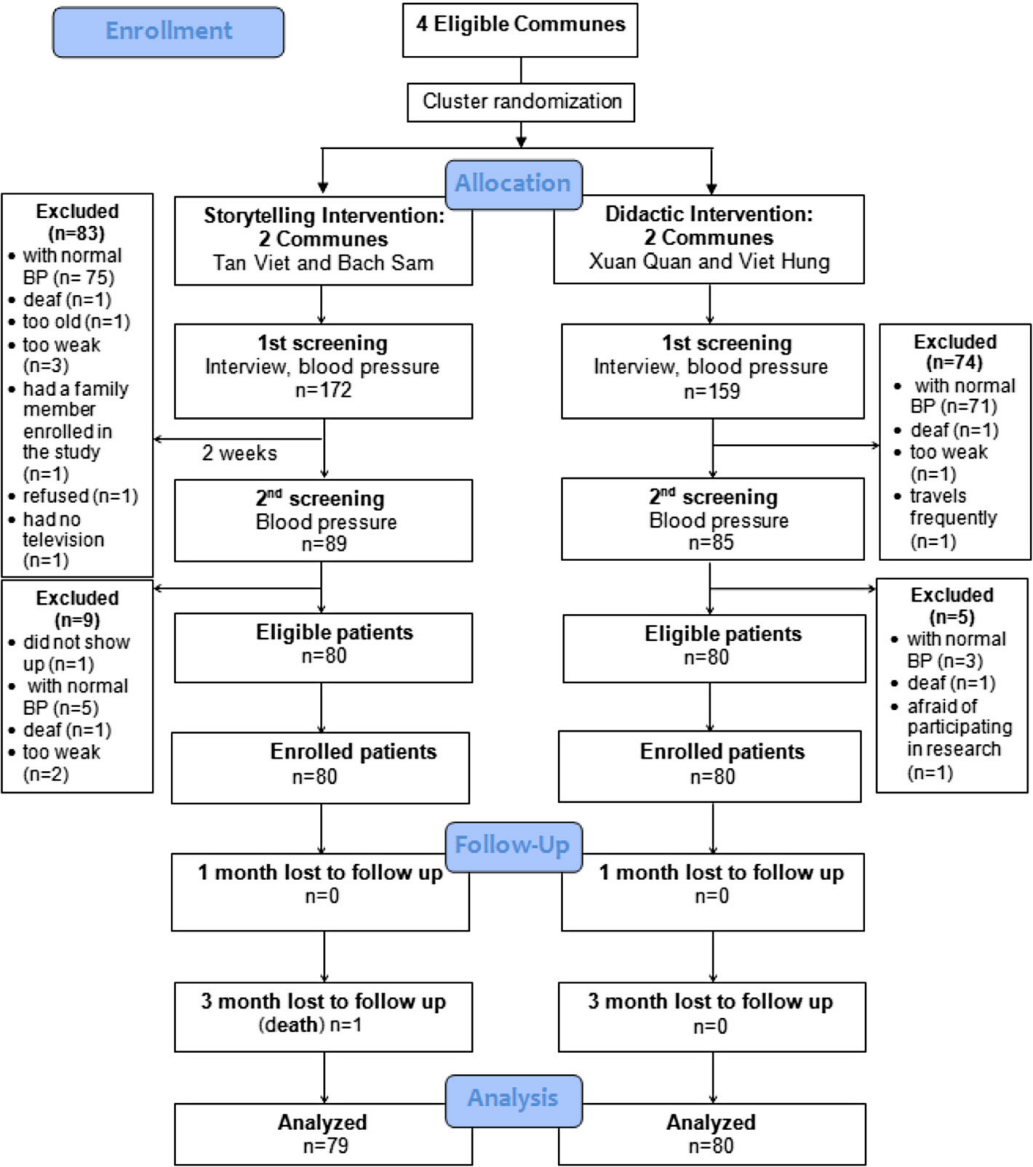
CONSORT flow diagram for the feasibility cluster-randomized controlled trial

## References

[1] Nguyen HL, Allison JJ, Ha DA, Germán C, Ly HN, Tran HT, Nguyen CK, Dang DM, Phan NT, Vu NC, Nguyen QP, Goldberg RJ (2017). Culturally adaptive storytelling intervention versus didactic intervention to improve hypertension control in Vietnam: a cluster-randomized controlled feasibility trial. Pilot and Feasibility Studies.

